# A Novel BODIPY-Derived Fluorescent Sensor for Sulfite Monitoring

**DOI:** 10.3390/s25206332

**Published:** 2025-10-14

**Authors:** Junyu Qu, Yixuan Liu, Wenqiang Fang, Huitao Liu, Zhenbo Liu

**Affiliations:** School of Chemistry and Chemical Engineering, Yantai University, Yantai 264005, China; 17762092728@163.com (J.Q.); liuyx2025@foxmail.com (Y.L.); f13954774039@163.com (W.F.)

**Keywords:** BODIPY, fluorescent probe, ethyl cyanoacetate, bisulfite radical

## Abstract

Sulfur dioxide (SO_2_) is commonly employed as an antioxidant and preservative in food processing, but excessive intake of SO_2_ can pose significant health risks. Therefore, accurate detection of sulfite content in food is crucial for ensuring food quality and safety. A novel fluorescent probe, BODIPY-Y, composed of a BODIPY derivative and an ethyl cyanoacetate group linked by a carbon–carbon double bond, was synthesized for detecting sulfur dioxide derivatives. When the BODIPY-Y probe interacts with SO_3_^2−^, the probe exhibits enhanced fluorescence at 514 nm. Spectrometric experiments show that the probe exhibits high sensitivity (*LOD*: 0.263 μmol/L), a fast response time (50 s) and excellent selectivity for SO_3_^2−^. Mechanistic studies confirm that the BODIPY-Y probe operates via an intramolecular charge transfer (ICT) mechanism. The carbon–carbon double bond in BODIPY-Y undergoes nucleophilic addition with SO_3_^2−^, blocking the ICT process and resulting in a blue shift in the fluorescence spectrum. In addition, the probe was applied to quantify SO_3_^2−^ levels in real food samples. The measured concentrations of SO_2_ in the white sugar and red wine were 15.93 μmol/L and 7.30 μmol/L, respectively, with recovery rates of 77.9–98.1%. This work presents a prospective chemical tool for monitoring sulfur dioxide derivatives in food products.

## 1. Introduction

Sulfur dioxide (SO_2_) is a major atmospheric pollutant, and predominantly emitted from industrial production processes [1,2]. Sulfur dioxide (SO_2_) typically exists as three main derivatives: sulfite, bisulfite, and sodium pyrosulfite [3,4]. Due to their reducibility, antibacterial activity and bleachability, these compounds are widely used for food preservation and color stabilization in products such as wine, sugar, and bean curd. At the same time, sulfur dioxide not only is an important endogenous gas transmitter [5,6], but also plays significant roles in many physiological activities including signal transduction, muscle expansion, and vasodilation [7,8]. However, excessive intake of sulfur dioxide may cause serious health hazards. Therefore, accurate detection of sulfur dioxide is crucial for environmental monitoring, food safety and life research.

There are numerous methods for detecting sulfur dioxide and its derivatives, including electrochemical analysis, titration, chromatography, and fluorescent probe techniques [9,10,11,12,13,14,15,16]. Among these, fluorescent probe technology is particularly advantageous due to fast response [17,18], low cytotoxicity [19,20], operational simplicity, good selectivity [21,22], high sensitivity [23,24] and real-time monitoring capability [25,26]. Consequently, fluorescent probes have been extensively utilized in chemical and biological analyses [27,28,29,30,31].

In recent years, the development of fluorescent probes for the detection of sulfur dioxide and its derivatives has been extremely rapid. Wu et al. [32] designed a PET mechanism-based fluorescent probe and reported a turn-on fluorescent probe (CMCA) for sulfite detection. The probe was synthesized via a condensation reaction between coumarin aldehyde and ethyl cyanoacetate, followed by a nucleophilic addition reaction with HSO_3_^−^. The probe has high specificity, excellent response kinetics and low detection limit for bisulfite. Based on the ICT effect, Du et al. [33] designed and synthesized a mitochondria-targeted fluorescent probe Mito-NPH, in which a strong electron-withdrawing 4-pyridinium acrylonitrile group was grafted onto an electron-donating naphthalene moiety. When HSO_3_^−^/SO_3_^2−^ is added to the probe Mito-NPH, the fluorescence signal of the probe varied from red to blue, and the response signal of HSO_3_^−^/SO_3_^2−^ was higher than other analytes. In addition, Mito-NPH demonstrates an ultra-fast response (within 10 s) to HSO_3_^−^. Meanwhile, cell imaging confirmed that the probe could target mitochondria and detect SO_2_. Ren et al. [34] designed a sulfite fluorescent probe NIR-TS based on the ESIPT mechanism The addition of sulfite induces changes the electron distribution of the benzopyridine unit in the probe, which enhances the ESIPT process, thereby generating near-infrared fluorescence. The probe was used to image SO_2_ derivatives in cells and mice. Liu et al. [35] built a near-infrared fluorescent probe (CQC) based on fluorescence resonance energy transfer (FRET) mechanism, composed of coumarin and quinoline fluorophore for the detection of SO_2_ derivatives. The probe CQC with a large Stokes shift (260 nm) not only ensures a sufficiently wide separation between the two emission peaks (165 nm), but also exhibits exceptionally high energy transfer efficiency (99.5%) and remarkable sensitivity for the detection of HSO_3_^−^/SO_3_^2−^ (*LOD* = 0.1 μmol/L). The effective probe CQC successfully visualized endogenous SO_3_^2−^/HSO_3_^−^ in living cells in real time. However, most of these fluorescent probes have high detection limits and long response times, making them unsuitable for rapid detection of low levels of sulfites in food (See Table S1). Therefore, it is necessary to develop a new SO_2_ fluorescent probe with fast response speed, strong mitochondrial targeting property, good biocompatibility and clear fluorescence changes.

Since their initial discovery by Treibs and Kreuzer in 1968 [36], BODIPY dyes have attracted increasing attention in the development of fluorescent probes due to their outstanding properties, including high sensitivity, exceptional stability under physiological conditions, minimal pH dependence, diverse modification sites, low cytotoxicity, and a remarkably high fluorescence quantum yield [37,38]. However, its relatively poor water solubility is one reason that limits its wide application in life research. Additionally, its Stokes displacement is small, and it is prone to self-quenching. These limitations restrict its application in fields such as biological imaging and the detection of endogenous substances in cells [39,40], while by modifying the structure of BODIPY and introducing different functional groups, the absorption and emission wavelengths of these dyes can be fine-tuned. This coordination enables the fluorescent properties of BODIPY derivatives to be tailored for variety of applications [41]. Therefore, we introduced an ethyl cyanoacetate group into BODIPY fluorophore to design an ICT-based fluorescence probe for sulfite detection, with the BODIPY fluorophores acting as the electron donors and ethyl cyanoacetate as the electron acceptor.

The objective of this study is to design and synthesize an ICT-based sulfur dioxide probe (BODIPY-Y), investigate its spectral properties, and explore its application for sulfite detection in food samples. This study is expected to offer novel insights for the development of BODIPY-based fluorescent probes for sulfur dioxide detection.

## 2. Materials and Methods

### 2.1. Material and Instrument

4-Formylbenzoic acid (>97%), boron trifluoride diethyl etherate (>98%), 2,4-dinitrophenylhydrazine (>98%), 2,4-dimethylpyrrole (>97%), N,N-dimethylformamide, triethylamine and trifluoroacetic acid (TFA) were purchased from Shanghai Macklin Biochemical Technology Co., Ltd. (Shanghai, China). 2,3-Dichloro-5,6-dicyano-p-benzoquinone (DDQ) (>97%) was purchased from Shanghai Aladdin Biochemical Technology Co., Ltd. (Shanghai, China). Phosphorus oxide trichloride, sodium bicarbonate, dichloromethane, acetic acid, ethyl acetate, hydrochloric acid, sodium sulphite, methanol and ethanol absolute were purchased from Sinopharm Group Chemical Reagent Co., Ltd. (Shanghai, China). The reagents employed in the experiment were all analytical grade and were utilized without further purification.

The types of the UV-VIS spectrophotometer and the fluorescence spectrophotometer are PERSEE UV-T700 and Cary Eclipse (Beijing, China), respectively. A Bruker Avance 400 spectrometer was used to record ^1^H NMR (Karlsruhe, Germany). A high-resolution mass spectrometer LCMS-IT-TOF confirmed the molecular weight of the compounds (Shimadzu, Kyoto, Japan). Preparation of PBS buffers of different pH values using a pH meter equipped with a saturated calomel electrode (Leici ZDJ-5B-G, Shanghai, China). Cell images were recorded using a laser scanning confocal microscope FV1200 (Olympus, Hamburg, Germany).

### 2.2. Synthesis of Compound ***1***

4-Formylbenzoic acid (1.32 g), 2,4-dimethylpyrrole (1.4 mL) and trifluoroacetic acid (0.3 mL) were added to a round-bottom flask, followed by 2,3-dichloro-5,6-dicyano-p-benzoquinone (1.96 g), and stirred under ice bath conditions. Add triethylamine and boron trifluoride etherate adjust reaction pH, and stirring in ice bath. After the completion of the reaction, it was separated by column chromatography with CH_2_Cl_2_/CH_3_OH (*v*/*v* = 10:1) as the eluent. The product was dried to a purplish-red solid, and Compound **1** was obtained with a yield of 35.71%. The reaction’s success was verified using ^1^H NMR spectroscopy (Figure S1) and Mass spectrum (Figure S2). ^1^H NMR (400 MHz, DMSO-d6) δ 8.10 (d, J = 8.2 Hz, 2H), 7.49 (d, J = 8.2 Hz, 2H), 6.20 (s, 2H), 2.46 (s, 6H), 1.34 (s, 6H); *m*/*z* = 367.15, it is consistent with the theoretical molecular weight of Compound **1** [M–H]^+^: 367.14.

### 2.3. Synthesis of Compound ***2***

N, N-dimethylformamide (10 mL) and phosphorus oxide trichloride (10 mL) were added to a three-necked flask, stirred in an ice bath, and stirred at room temperature for 0.5 h. Then the Compound **1** dissolved in dichloromethane was added and stirred at 75 °C for 6 h under N_2_ conditions. After the reaction is over, add saturated sodium bicarbonate solution and stir until there are no bubbles. After extraction and separation, anhydrous Na_2_SO_4_ was added to dry and the solvent was removed. The purplish red solid was purified by CH_2_Cl_2_/MeOH (*v*/*v* = 100:6) silica gel column, and the Compound **2** was obtained with yield of 56.06. ^1^H NMR (400 MHz, Chloroform-d, Figure S3) δ 10.02 (s, 1H), 7.63 (d, J = 8.1 Hz, 2H), 7.38 (d, J = 8.1 Hz, 2H), 6.19 (s, 1H), 2.84 (s, 3H), 2.64 (s, 3H), 1.69 (s, 3H), 1.48 (s, 3H); *m*/*z* = 395.09 (Figure S4), it is consistent with the theoretical molecular weight of Compound **2** [M–H]^+^: 395.15.

### 2.4. Synthesis of the Fluorescent Probe BODIPY-Y

Compound **2**, triethylamine and ethyl cyanoacetate were added to the three-port flask and the solution was stirred back for 4 h. After the reaction, the solvent was removed, and the crude product was separated and purified by CH_2_Cl_2_/MeOH (*v*/*v* = 100:6) silica gel column to obtain a dark red solid BODIPY-Y with a yield of 56.65%. ^1^H NMR (400 MHz, Chloroform-d, Figure S5) δ 8.29 (d, J = 8.0 Hz, 2H), 8.16 (s, 1H), 7.48 (d, J = 8.2 Hz, 2H), 6.16 (s, 1H), 4.35 (q, J = 7.1 Hz, 2H), 2.66 (s, 3H), 2.63 (s, 3H), 1.46 (s, 3H), 1.42 (s, 3H), 1.38 (t, J = 7.2 Hz, 3H); *m*/*z* = 490.18 (Figure S6), it is consistent with the theoretical molecular weight of probe BODIPY-Y [M–H]^+^: 490.3.

### 2.5. Fluorescence Measurement Conditions

Preparation of the probe BODIPY-Y was prepared to 0.5 mmol/L, and other concentrations were attained by adding methanol. Various interfering anionic and cationic solutions were made up in deionized water. The working solution was PBS buffer (10 mmol/L, pH = 7.4). The instrument parameters include 2.5 nm excitation slit, 5 nm emission slit, 480 nm excitation wavelength, 490–580 nm detection range and 700 V photomultiplier tube voltage.

### 2.6. Cell Cytotoxic and Imaging

Hela cells were cultivated overnight in a complete cell medium with a ratio of MEM:FBS of 9:1 in a 5% CO_2_ incubator at 37 °C.

Cytotoxicity was tested by MTT method. Hela cells in logarithmic growth phase were inoculated at 6 × 10^3^ cells per well into 96-well plates and cultured for 24 h. The medium was removed and each well was rinsed three times with PBS. 100 μL of medium including 0.5 mg/mL MTT was added to each well, and incubated at 37 °C for 4 h in a constant temperature with 5% CO_2_. The supernatant was dumped and 100 μL DMSO was pipetted into each of the well. After oscillating for 10 min, the absorbance at 570 nm was determined.

A laser confocal microscope was used to observe the imaging of the samples in the cells. Hela cells in logarithmic growth phase were cultured overnight by inoculating them into laser confocal dishes at a ratio of 6 × 10^4^ cells per well. The sample was aroused by a 488 nm laser, and the experimental results were observed and recorded under a 10-fold eyepiece and a 100-fold objective.

## 3. Results

### 3.1. Synthesis of the Probe BODIPY-Y

**Scheme 1:**
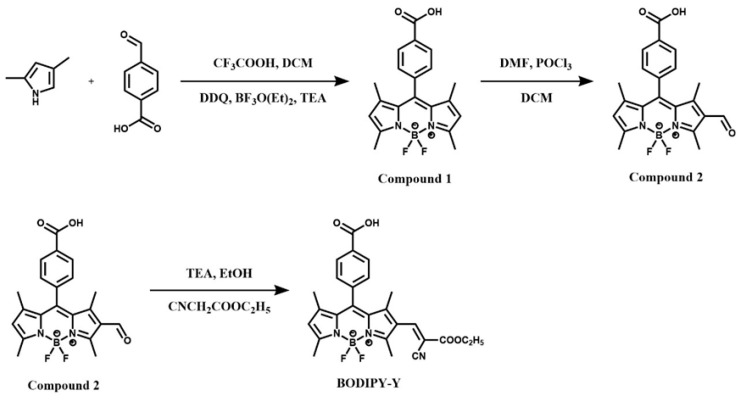
The synthetic route of BODIPY-Y fluorescent probe.

The full synthetic route of probe BODIPY-Y is displayed in Scheme 1. The satisfactory spectral data, including ^1^H NMR and MS, confirmed the structure of the compound (Figures S1–S6).

### 3.2. Spectral Characteristics of Probe BODIPY-Y for SO_3_^2−^

**Figure 1:**
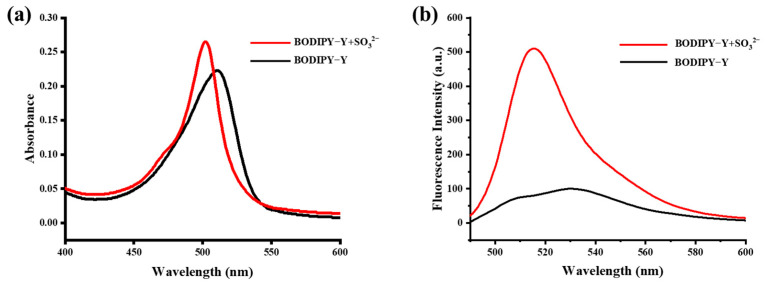
(**a**) UV-Vis absorption spectra of probe BODIPY-Y prior to and after addition of SO_3_^2−^. (**b**) Fluorescence spectra of probe BODIPY-Y prior to and after addition of SO_3_^2−^.

To explore the spectral features of probe BODIPY-Y toward SO_3_^2−^, we studied the UV-vis and fluorescence spectra of the interaction, as shown in Figure 1.

From the UV absorption spectrum of the probe BODIPY-Y solution in Figure 1a, it can be clearly seen that probe BODIPY-Y has a prominent UV absorption peak with a maximum absorption at 510 nm. The addition of SO_3_^2−^ narrowed the ultraviolet spectra of the solution, and the maximum absorption wavelength was bathochromic-shift to 502 nm, indicating that the probe reacted with SO_3_^2−^ to break the old conjugated structure. It can be watched with the unaided eye that the color changes from orange to yellow-green. When SO_3_^2−^ was added into probe BODIPY-Y solution, the enhancement of the fluorescence signal was very remarkable, and the fluorescence color changed from bright yellow to strong green. It shows that the probe can be used for the detection of SO_3_^2−^.

### 3.3. Sensitivity of Probe BODIPY-Y to SO_3_^2−^

**Figure 2:**
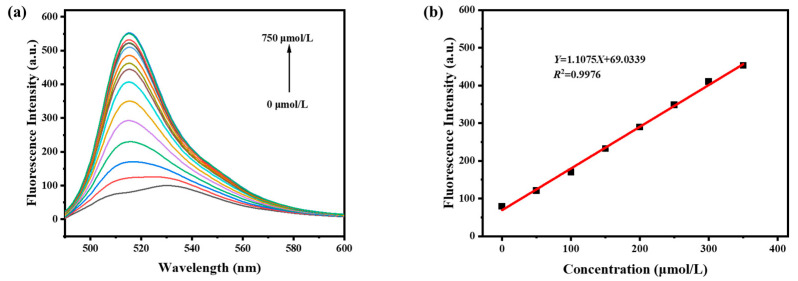
(**a**) Fluorescence emission spectra of probe and different concentrations of SO_3_^2−^ (0–750 μmol/L). (**b**) The linear relationship between the concentration of SO_3_^2−^ and the fluorescence intensity was obtained.

The linear relationship between the fluorescence probe and the SO_3_^2−^ concentration was studied. At room temperature, in the ethanol/PBS (*v*/*v*, 1/9) buffer solution system, the SO_3_^2−^ solution was quantitatively added to the fluorescent probe solution, and the intensity was measured by a fluorescence spectrophotometer. As shown in Figure 2a, the fluorescence intensity at 514 nm gradually increased with the rising of SO_3_^2−^ concentration. In addition, the emission spectrum of the solution exhibited a blue-shifted along with a significant enhancement in intensity.

In the range of 0–350 μmol/L, the relationship between fluorescence intensity and SO_3_^2−^ concentration was linear and calculated as *Y* = 69.0339 + 1.1073*X*, *R*^2^ = 0.9976 (Figure 2b). The fluorescence intensity of 11 groups of blank fluorescent probe solutions without SO_3_^2−^ was also measured. The standard deviation of the fluorescence intensity at 514 nm was calculated to be 0.0877, and the limit of detection (*LOD*) was estimated to be 0.263 μmol/L by the formula $\mathrm{LOD}=\frac{3\sigma }{k}$ (“*k*” represents the slope of the standard curve, and “*σ*” is the standard deviation of the blank sample).

### 3.4. Stability and Reactivity of Probe BODIPY-Y on SO_3_^2−^

To explore the stability of the probe BODIPY-Y in the buffer solution, the effect of pH on its fluorescence intensity was investigated. The probe was prepared in buffer solutions of different pH and then used to detect SO_3_^2−^.

**Figure 3:**
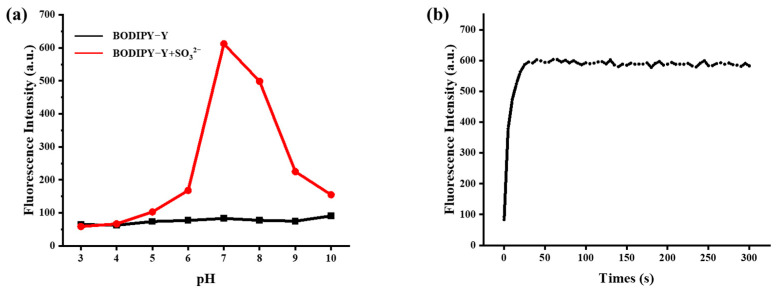
(**a**) The influences of pH on the fluorescence intensity of the probe. (**b**) The fluorescence kinetic curve of the interaction between fluorescent probe and SO_3_^2−^.

From Figure 3a, the fluorescence response of the probe to sodium sulfite exhibited strong pH dependence. The fluorescence intensity of the fluorescent probe to sodium sulfite was weak when the pH was less than 3.0 or more than 10.0, suggesting limited applicability in strong acids or bases environments. In the range of pH 5.0–8.0, the fluorescence intensity increased prominently, and the fluorescence intensity of the probe to sodium sulfite was the highest at pH 7.0, indicating that the probe is most suitable for SO_3_^2−^ detection under neutral conditions.

One of the advantages of fluorescent probes compared to other detection methods is the ability to detect quickly. For the purpose of investigating the responsiveness of the probe BODIPY-Y to SO_3_^2−^, the probe’s response time to react with SO_3_^2−^ was measured. The quartz cuvette was added 3 mL of a fluorescent probe solution with a concentration of 5 μmol/L, followed by transferring 3 μL of a 50 mmol/L sodium SO_3_^2−^ solution in the cuvette and carefully stirring the probe solution to mix evenly. The fluorescence intensity of the fluorescent probe at 514 nm was detected by a fluorescence spectrophotometer, and the relationship between the time variation and the fluorescence intensity change was obtained. From Figure 3b, it can be seen that the fluorescent probe interacts with SO_3_^2−^ quickly, and the spectral intensity reaches the peak within 50 s and is steady after 100 s. It shows that the probe has a rapid response speed and excellent photostability to SO_3_^2−^.

### 3.5. Selective Response of Probe BODIPY-Y to SO_3_^2−^

**Figure 4:**
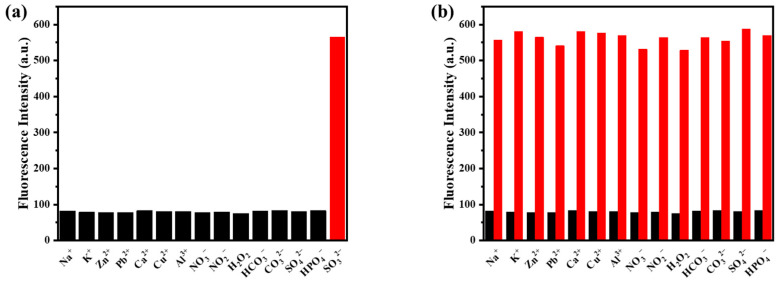
(**a**) Fluorescent probe selective detection curve. (**b**) Fluorescent probe selective detection of fluorescence intensity histogram.

The selectivity of the probe toward SO_3_^2−^ was systematically investigated, as illustrated in Figure 4a. Upon addition of 2 mmol/L SO_3_^2−^, the probe showed a notable fluorescence enhancement roughly 5-fold increase. In contrast, the characteristic fluorescence intensity of the solution remained nearly unchanged after adding 2 mmol/L of representative ions (Na^+^, K^+^, Zn^2+^, Pb^2+^, Ca^2+^, Cu^2+^, Al^3+^, NO_3_^−^, NO_2_^−^, H_2_O_2_, HCO_3_^−^, CO_3_^2−^, SO_4_^2−^, HPO_4_^2−^) compared to the blank sample. This remarkable phenomenon indicates that the probe only shows a specificity to SO_3_^2−^, confirming the ability of the probe to accurately detect SO_3_^2−^ in complex samples.

Competitive binding experiments further confirmed the practical applicability of the probe (Figure 4b). The black bars represent the fluorescence emission intensity produced when the probe was exposed to each interfering ion separately, while the red bars show the fluorescence intensity after subsequent addition of SO_3_^2−^. The results indicated that the co-existing ions exhibited negligible interference with the fluorescence emission triggered by SO_3_^2−^, confirming the BODIPY-Y probe’s high anti-interference capability for SO_3_^2−^. The results revealed the application potential of the probe BODIPY-Y in environmental monitoring and food safety.

### 3.6. Reaction Mechanism

**Figure 5:**
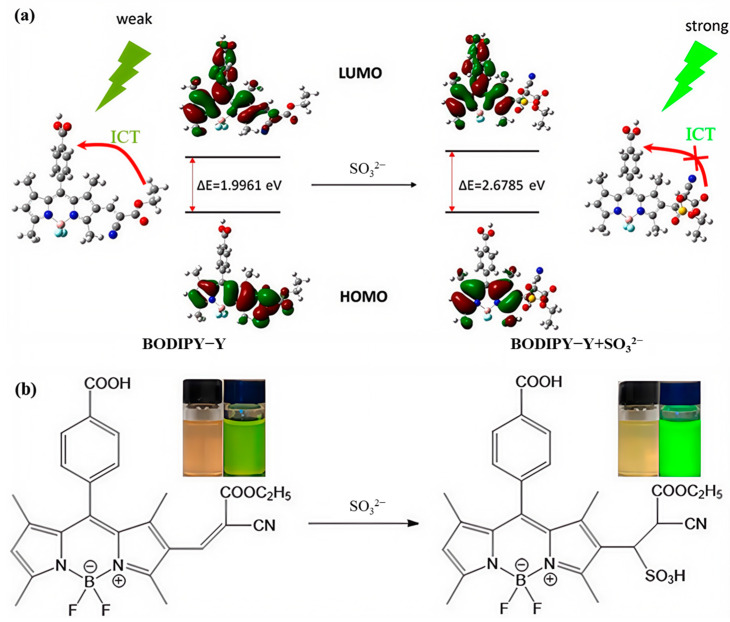
(**a**) The molecular orbitals of BODIPY-Y. (**b**) The reaction mechanism of probe BODIPY-Y.

The photochemical properties and its detection mechanism of probe BODIPY-Y can be well illustrated by using frontier molecular orbitals (FMOs) theory and Time-Dependent Density Functional Theory (TDDFT) calculations. To further validate the response mechanism of the fluorescent probe, we used GAUSSIAN 09 [42] software to correct and calculate the HOMO-LUMO gap between lowest unoccupied molecular orbital (LUMO) and highest occupied molecular orbital (HOMO) in two configurations. The BODIPY-Y probe structure consists of a BODIPY derivative group and an ethyl cyanoacetate group connected by a carbon–carbon double bond. The BODIPY-Y probe operates via an intramolecular charge transfer (ICT) from an ethyl cyanoacetate group to BODIPY derivative group, as evidenced by the HOMO and LUMO distribution in Figure 5a. The ICT process results in low emission intensity of the probe. When the probe reacts with SO_3_^2−^, the structure BODIPY-Y + SO_3_^2−^ is formed, which was also confirmed by mass spectrometry displaying a molecular ion peak at *m/z* 572.15 in good agreement with the theoretical value of 572.37 (Figure S7). This nucleophilic addition cleaves the critical C=C bond between the BODIPY core and ethyl cyanoacetate moiety, thereby disrupting the ICT process. As a result, the system exhibits strong fluorescence emission. In addition, the BODIPY-Y probe exhibits a narrower HOMO-LUMO gap (1.9961 eV) compared to BODIPY-Y + SO_3_^2−^ (2.6786 eV) correlating well with the significant emission blueshift observed upon SO_2_ interaction in Figure 1b.

### 3.7. Cytotoxicity

**Figure 6:**
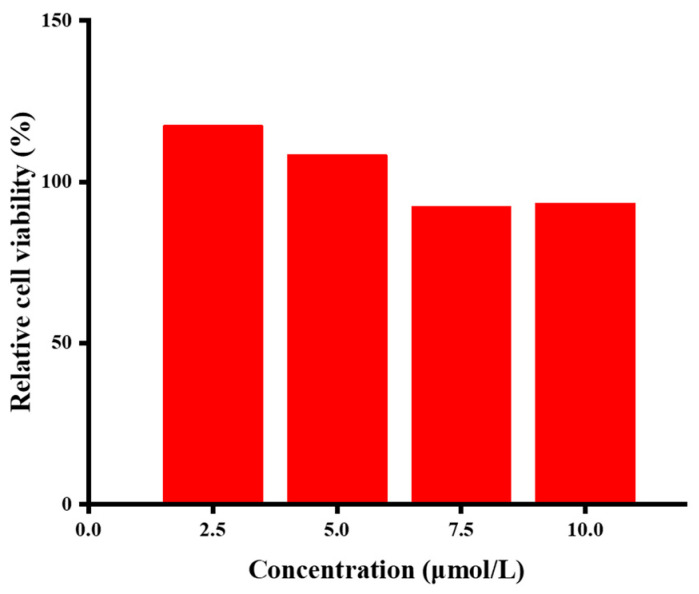
The cytotoxicity of different concentrations of BODIPY-Y on Hela cells was determined.

Cytotoxicity represents a critical consideration in live-cell imaging applications. To establish optimal imaging conditions while preserving cellular viability, rigorous assessment of probe cytotoxicity is imperative. In this experiment, Hela cells were used to evaluate the toxicity of BODIPY-Y. As shown in Figure 6, more than 90% of the cells were alive at 10 μmol/L BODIPY-Y. Therefore, BODIPY-Y imaging is non-toxic to living cells. 

### 3.8. Biological Imaging

**Figure 7:**
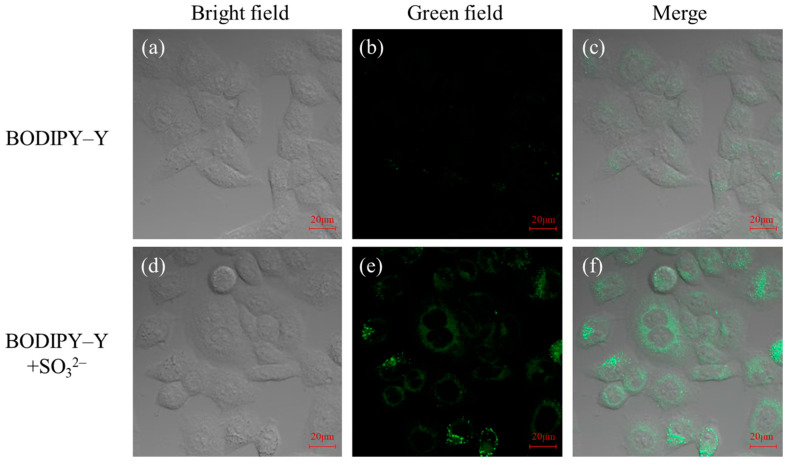
Confocal images of HeLa cells under different incubation conditions. (**a**–**c**) Control group: incubated with probe BODIPY-Y (5 μM) for 60 min. (**d**–**f**) Incubated with sulfite (500 μM) for 30 min, followed by incubation with probe BODIPY-Y (5 μM) for 30 min. Incubation temperature: 37 °C.

Intracellular imaging of live HeLa cells was performed to assess the response of BODIPY-Y to sulfite. As shown in Figure 7, in control experiments, incubation with the BODIPY-Y probe (5 μM) at 37 °C for 60 min yielded a faint green fluorescence signal. Subsequent treatment with sulfite (500 μM) for an additional 30 min at 37 °C resulted in a marked enhancement of this fluorescence. These findings demonstrate that BODIPY-Y is capable of monitoring dynamic changes in intracellular sulfite levels.

### 3.9. Detection of SO_3_^2−^ in Food

In an effort to study the application of BODIPY-Y in actual samples, fluorescence spectroscopy was used to detect the concentrations of sodium sulfite in white cane sugar and red wine, as shown in Table 1. Herein, white cane sugar and red wine were reconstituted in ultrapure water to create an aqueous solution for subsequent use. The sulfite content in the two food samples, as determined experimentally, was found to be 15.93 μmol/L and 7.30 μmol/L, respectively. To assess the accuracy of the measurement, sulfites of known concentrations were added to these samples and detected using probes. The sulfite content detected by the probe showed good agreement with the spiked concentrations, demonstrating recovery rates ranging from 77.9% to 98.1%. This indicates that the probe has good reliability and accuracy in sulfite analysis in actual food samples, confirming its application potential in the field of food analysis.

## 4. Conclusions

In summary, we have developed a novel ICT-based fluorescence probe, BODIPY-Y, for detecting sulfur dioxide derivatives. The probe successfully identifies sulfites in food samples such as sugar and red wine. Furthermore, BODIPY-Y exhibits extraordinary sensitivity and rapid response time, making it a promising candidate for the detection of sulfur dioxide (SO_2_) derivatives. Notably, BODIPY-Y shows superior selectivity and strong anti-interference capability, suggesting its strong potential for reliable SO_3_^2−^ monitoring in complex matrices.

## Figures and Tables

**Table 1:** Detection results of probe BODIPY-Y in food samples.

Samples	Na_2_SO_3_ Spiked (μmol/L)	Found Mean (μmol/L)	Recovery (%)	RSD (%)
White sugar	0	15.93	-	-
	25	35.4	77.9	0.7
	50	62.23	93.6	1.4
	75	89.53	98.1	1.4
Red wine	0	7.3	-	-
	25	30.79	94.0	4.9
	50	52.33	90.1	1.6
	75	71.11	85.1	1.2

## Data Availability

The data are contained within the article.

## Supplementary Materials

The following supporting information can be downloaded at: https://www.mdpi.com/article/10.3390/s25206332/s1, Figure S1: ^1^H NMR of the Compound **1**; Figure S2: Mass spectrum of Compound **1**; Figure S3: ^1^H NMR of the Compound **2**; Figure S4: Mass spectrum of Compound **2**; Figure S5: ^1^H NMR of the fluorescent probe BODIPY-Y; Figure S6: Mass spectrum of the fluorescent probe BODIPY-Y; Figure S7: Mass spectrum of BODIPY-Y after reaction with SO_3_^2−^; Table S1: Comparison of fluorescent probes for SO_3_^2−^. Refs. [43,44,45,46,47,48,49,50,51,52] cited in Supplementary Materials.

## References

[1] Zhang S.-Y., Yang X.-P., Xu Y., Wang H.-Y., Luo F., Fu G.-M., Yan D.-W., Lai M., Ke Y., Ye Y. (2024). Rational design of a rapidly responsive and highly selective fluorescent probe for SO_2_ derivatives detection and imaging. Food Chem..

[2] Cui W.-L., Wang M.-H., Yang Y.-H., Ji X., Wang J.-Y. (2023). Viscosity & SO_2_-sensitive dual colorimetric effect fluorescent sensor enabled imaging detection within plant onion and biological system. Spectrochim. Acta A Mol. Biomol. Spectrosc..

[3] Tian L., Sun X.-F., Zhou L.-F., Zhong K.-L., Li S.-D., Yan X.-M., Tang L.-J. (2023). Reversible colorimetric and NIR fluorescent probe for sensing SO_2_/H_2_O_2_ in living cells and food samples. Food Chem..

[4] D’Amore T., Di Taranto A., Berardi G., Vita V., Marchesani G., Chiaravalle A.E., Iammarino M. (2020). Sulfites in meat: Occurrence, activity, toxicity, regulation, and detection. A comprehensive review. Compr. Rev. Food Sci. Food Saf..

[5] Luo L.-M., Chen S., Jin H.-F., Tang C.-S., Du J.-B. (2011). Endogenous generation of sulfur dioxide in rat tissues. Biochem. Biophys. Res. Commun..

[6] Huang Y.-Q., Zhang H., Lv B.-Y., Tang C.-S., Du J.-B., Jin H.-F. (2021). Sulfur Dioxide: Endogenous Generation, Biological Effects, Detection, and Therapeutic Potential. Antioxid. Redox Signal..

[7] Meng Z.-Q., Yang Z.-H., Li J.-L., Zhang Q.-X. (2012). The vasorelaxant effect and its mechanisms of sodium bisulfite as a sulfur dioxide donor. Chemosphere.

[8] Khalaf E.M., Mohammadi M.J., Sulistiyani S., Ramírez-Coronel A.A., Kiani F., Jalil A.T., Almulla A.F., Asban P., Farhadi M., Derikondi M. (2024). Effects of sulfur dioxide inhalation on human health: A review. Rev. Environ. Health.

[9] Zhao X.-Y., Zhang Y.-J., Han X., Qi H.-C., Ma F.-X., Chen K. (2024). Pressure-Compensated Fiber-Optic Photoacoustic Sensors for Trace SO_2_ Analysis in Gas Insulation Equipment. Anal. Chem..

[10] Zhao L., Zhou J.-X., Zhou J., Lin X.-J., Huang K., Jiang X., Yu H.-M., Xiong X.-L. (2022). A microplasma converter-based spectrophotometry and visual colorimetry for nonchromatographic speciation analysis of H_2_S/SO_2_ or S^2−^/SO_3_^2−^ in environmental water samples. Microchem. J..

[11] Zhang S.-Y., Lai M., Gao Z.-T., Li H.-X., Yang X.-P., Wei Y.-W., Luo F., Yu Z.-J., Zhang D., Ji X.-M. (2023). An ICT-based ratiometric fluorescent probe for tracking and imaging SO_2_ during Cd-induced stress in plants. Sens. Actuators B Chem..

[12] Yang Y.-B., Li J.-H., Zhang Z.-H., Wang J.-N., Lin G.-Y. (2023). Quantitative analysis of SO_2_, NO_2_ and NO mixed gases based on ultraviolet absorption spectrum. Analyst.

[13] Wen X.-Y., Li F., Liu F.-R., Fan Z.-F. (2022). A novel ratiometric sensor prepared based aggregation-induced emission for ultrafast detection of SO_2_ derivatives in food samples and living cells. Anal. Chim. Acta.

[14] Prasertying P., Ninlapath T., Jantawong N., Wongpakdee T., Sonsa-ard T., Uraisin K., Saetear P., Wilairat P., Nacapricha D. (2022). Disposable Microchamber with a Microfluidic Paper-Based Lid for Generation and Membrane Separation of SO_2_ Gas Employing an In Situ Electrochemical Gas Sensor for Quantifying Sulfite in Wine. Anal. Chem..

[15] Gajdár J., Herzog G., Etienne M. (2022). Amperometric Sensor for Selective On-Site Analysis of Free Sulfite in Wines. ACS Sens..

[16] Fang Y.-Y., Zheng D.-B., Zhang T.-R., Cao Z.-X., Zhou H.-C., Deng Y., Peng C. (2024). A rationally designed fluorescent probe for sulfur dioxide and its derivatives: Applications in food analysis and bioimaging. Anal. Bioanal. Chem..

[17] Liu X.-N., Shi T.-Z., Xu C.-Y., Zhu M.-Q., Wang Y. (2023). A highly selective and sensitive ICT-based Cu^2+^ fluorescent probe and its application in bioimaging. Ecotoxicol. Environ. Saf..

[18] Cao X.-Y., Lu H.-Z., Wei Y.-F., Jin L.-X., Zhang Q., Liu B. (2022). A simple “turn-on” fluorescent probe capable of recognition cysteine with rapid response and high sensing in living cells and zebrafish. Spectrochim. Acta A Mol. Biomol. Spectrosc..

[19] Wang H.-F., Wang P.-P., Niu L.-F., Liu C.-H., Xiao Y.-Z., Tang Y., Chen Y. (2022). Carbazole-thiophene based fluorescent probe for selective detection of Cu^2+^ and its live cell imaging. Spectrochim. Acta A Mol. Biomol. Spectrosc..

[20] Hou S.-M., Wang Y., Zhang Y.-K., Wang W.-J., Zhou X. (2022). A reversible turn-on fluorescent probe for quantitative imaging and dynamic monitoring of cellular glutathione. Anal. Chim. Acta.

[21] Zhou B.-X., Wang B.-B., Bai M.-Q., Dong M.-D., Tang X. (2023). Fluorescent probe for highly selective detection of cysteine in living cells. Spectrochim. Acta A Mol. Biomol. Spectrosc..

[22] Xu Z.-L., Liu S.-T., Xu L.-R., Li Z.-C., Zhang X.-L., Kang H., Liu Y.-F., Yu J., Jing J., Niu G.-L. (2024). A novel ratiometric fluorescent probe with high selectivity for lysosomal nitric oxide imaging. Anal. Chim. Acta.

[23] Peng J.-L., Ju Q.-K., An B.-S., Yin Z.-J., Wei N.-N., Zhang Y.-R. (2022). A super sensitive fluorescent probe for imaging endogenous hydrogen sulfide in living cells. Talanta.

[24] Liang Z.-Y., Wei N., Guo X.-F., Wang H. (2023). A new quinoline based probe with large Stokes shift and high sensitivity for formaldehyde and its bioimaging applications. Anal. Chim. Acta.

[25] Yang K.-Q., Tang H.-D., Zhang Y.-P., Wu Y., Su L.-C., Zhang X., Du W., Zhang J.-P., Wang G.-Y., Liu D.-J. (2024). NIR-II Ratiometric Fluorescent Nanoplatform for Real-Time Monitoring and Evaluating Cancer Sonodynamic Therapy Efficacy. Adv. Opt. Mater..

[26] Tang L.Y., Li P.P., Han Y.Y., Yang G.Y., Xin H.T., Zhao S.F., Guan R.F., Liu Z.Q., Cao D.X. (2023). A fluorescein-based fluorescent probe for real-time monitoring hypochlorite. J. Photochem. Photobiol. A Chem..

[27] Zhou Z.-L., Fang C., Yu F.-J., Shen Y.-M., Xu H., Li H.-T., Zhang Y.-Y. (2024). Visualization of cysteine in AD mouse with a high-quantum yield NIR fluorescent probe. Talanta.

[28] Zhang Y.-B., Qiao L.-L., Zhang Z.-Q., Liu Y.-F., Li L.-S., Shen H.-B., Zhao M.-X. (2022). A mitochondrial-targetable fluorescent probe based on high-quality InP quantum dots for the imaging of living cells. Mater. Des..

[29] Wang Y., Li J.-H., Pei Z.-C., Pei Y.-X. (2022). Lactosylation leads to a water-soluble fluorescent probe for detection of S^2−^ in water. Microchem. J..

[30] Song J., Yu J.-Y., Sun K., Chen Z.-X., Xing X.-X., Yang Y.-M., Sun C.-Y., Wang Z.-F. (2023). A high quantum yield xanthene-based fluorescent probe for the specific detection of tyrosinase and cell imaging. J. Photochem. Photobiol. A Chem..

[31] Hu N., Zeng H., Shi S., Yao W., Ji D., Guo H., Luo L., Jin T., Yu Q., Xu K. (2023). The preparation of a chitosan-based novel fluorescent macromolecular probe and its application in the detection of hypochlorite. Mater. Today Chem..

[32] Wu J.-J., Pan J., Ye Z., Zeng L.-T., Su D.-D. (2018). A smart fluorescent probe for discriminative detection of hydrazine and bisulfite from different emission channels. Sens. Actuators B Chem..

[33] Du Y.-T., Pan C.-X., Cao C.-J. (2023). A mitochondria-targetable fluorescent probe for sulfur dioxide detection and visualisation in living cells. Spectrochim. Acta A Mol. Biomol. Spectrosc..

[34] Ren H.-X., Huo F.-J., Wu X., Liu X.-G., Yin C.-X. (2021). An ESIPT-induced NIR fluorescent probe to visualize mitochondrial sulfur dioxide during oxidative stress in vivo. Chem. Commun..

[35] Liu F.-T., Li N., Chen Y.-S., Yu H.-Y., Miao J.-Y., Zhao B.-X. (2022). A quinoline-coumarin near-infrared ratiometric fluorescent probe for detection of sulfur dioxide derivatives. Anal. Chim. Acta.

[36] Treibs A., Kreuzer F.-H. (1968). Difluorboryl-Komplexe von Di- und Tripyrrylmethenen. Justus Liebigs Ann. Chem..

[37] Poddar M., Misra R. (2020). Recent advances of BODIPY based derivatives for optoelectronic applications. Coordin. Chem. Rev..

[38] Das S., Dey S., Patra S., Bera A., Ghosh T., Prasad B., Sayala K.D., Maji K., Bedi A., Debnath S. (2023). BODIPY-Based Molecules for Biomedical Applications. Biomolecules.

[39] Yan M.-M., He D.-M., Zhang L.-S., Sun P.-J., Sun Y.-Q., Qu L.-B., Li Z.-H. (2022). Explorations into the meso-substituted BODIPY-based fluorescent probes for biomedical sensing and imaging. TrAC Trends Anal. Chem..

[40] Liu Y., Wu Y.-X., Zhang D.-L., Xi Y., Yu S.-R., Zhong H.-M., He K.-D., Li D., Wei W.-T., Cao Y.-T. (2020). A BODIPY-Hemicyanine-Based Water-Soluble Dual-Color Fluorescence Probe for Colorimetric Monitoring of Intracellular Endogenous Sulfur Dioxide and Bioimaging Applications. ChemistrySelect.

[41] Krumova K., Cosa G. (2010). Bodipy Dyes with Tunable Redox Potentials and Functional Groups for Further Tethering: Preparation, Electrochemical, and Spectroscopic Characterization. J. Am. Chem. Soc..

[42] Frisch M.J., Trucks G.W., Schlegel H.B., Scuseria G.E., Robb M.A., Cheeseman J.R., Scalmani G., Barone V., Mennucci B., Petersson G.A. (2010). Gaussian 09.

[43] Yang L., Yang N., Gu P., Zhang Y., Gong X., Zhang S., Li J., Ji L., He G. (2022). A novel naphthalimide-based fluorescent probe for the colorimetric and ratiometric detection of SO_2_ derivatives in biological imaging. Bioorg. Chem..

[44] Li X.-H., Yan J.-L., Wu W.-N., Zhao X.-L., Wang Y., Fan Y.-C., Xu Z.-H. (2022). A dual-response fluorescent probe for SO_2_ and viscosity and imaging application in lysosomes and zebrafish. Microchem. J..

[45] Zhong K., Li Y., Zhou L., Sun X., Tang L., Zhang N., Tang Y. (2024). A benzopyran-hemicyanine-based mitochondria-targeted NIR fluorescent probe for detection of SO_2_ derivatives in food samples and living cells. Microchem. J..

[46] Dutta K., Chattaraj S., Pal R., Jonnalgadda P.N., Patro B.S., Mula S., Chakraborty G. (2024). A Kumujian-C based highly selective fluorescence turn-on probe enables the detection of sulfite in real samples and living cells. New J. Chem..

[47] Liu F.-T., Han W.-W., Ren H., Wang R.-N., Yang W.-J., Miao J.-Y., Zhao B.-X., Lin Z.-M. (2023). A dicyanoisophorone-quinolinium-based near-infrared-emission fluorescent probe for ratiometric sensing of bisulfite/sulfite in living cells. Sens. Actuators B Chem..

[48] Cui R., Gao Y., Ge H., Shi G., Li Y., Liu H., Ma C., Ge Y., Liu C. (2022). A turn-on fluorescent probe based on indolizine for the detection of sulfite. New J. Chem..

[49] Li Y., Sun X., Zhou L., Tian L., Zhong K., Zhang J., Yan X., Tang L. (2022). Novel Colorimetric and NIR Fluorescent Probe for Bisulfite/Sulfite Detection in Food and Water Samples and Living Cells Based on the PET Mechanism. J. Agric. Food Chem..

[50] Huang Y., Xin H., Lin Q., Yang G., Zhang Y., Cao D., Yu X. (2024). A fluorescent probe for detecting bisulfite/sulfite in lipid droplets and tracking the dynamics of lipid droplets. Talanta.

[51] Liang T., Liu S., Shen T., Chen X., Li X., Yan X., Sun X., Tian M., Wu C., Sun X. (2024). Chromene-derived red-fluorescent probes for sulfite detection in food and living cells based on an integrated ICT&PET platform. Sens. Actuators B Chem..

[52] Yang W., Fang X., Chen C., Zhang Y., Zhang W., Qian J. (2024). Coumarin-based fluorescent probes for colorimetric and ratiometric fluorescent detection of sulfite: Structure-activity relationship. Tetrahedron.

