# Supplementary material for: A Novel BODIPY-Derived Fluorescent Sensor for Sulfite Monitoring
Source: Sensors (Basel). 2025 Oct 14;25(20):6332. doi: 10.3390/s25206332 (PMC12567704; doi:10.3390/s25206332)
Supplement: Supplementary file 1 [file sensors-25-06332-s001.zip › sensors-3841466-supplementary.pdf]

A Novel BODIPY-Derived Fluorescent Sensor for Sulfite Monitoring

Junyu Qu, Yixuan Liu, Wenqiang Fang, Huitao Liu * and Zhenbo Liu *

School of Chemistry and Chemical Engineering, Yantai University, Yantai 264005, China; 17762092728@163.com (J.Q.); liuyx2025@foxmail.com (Y.L.); 1297522420@qq.com (W.F.)
* Correspondence: liuht-ytu@163.com (H.L.); zhenboliu@foxmail.com (Z.L.)

1. ^{1}H NMR of the Compound 1

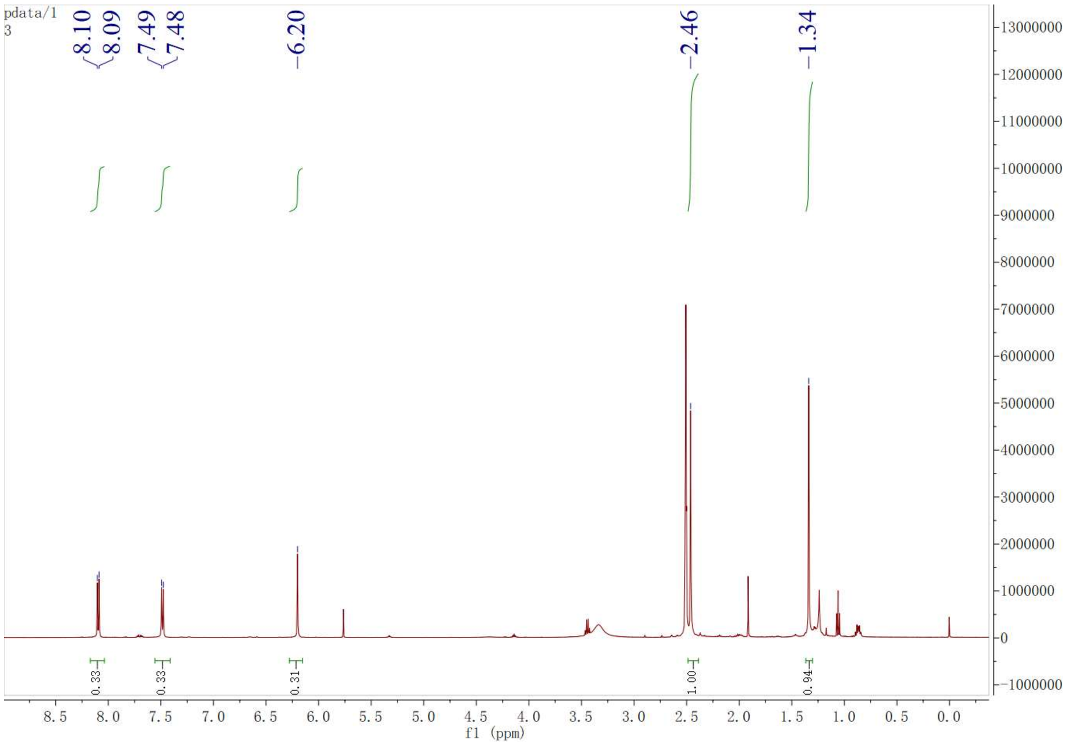

Figure S1 ^{1}H NMR of the Compound 1

2. Mass spectrum of Compound 1

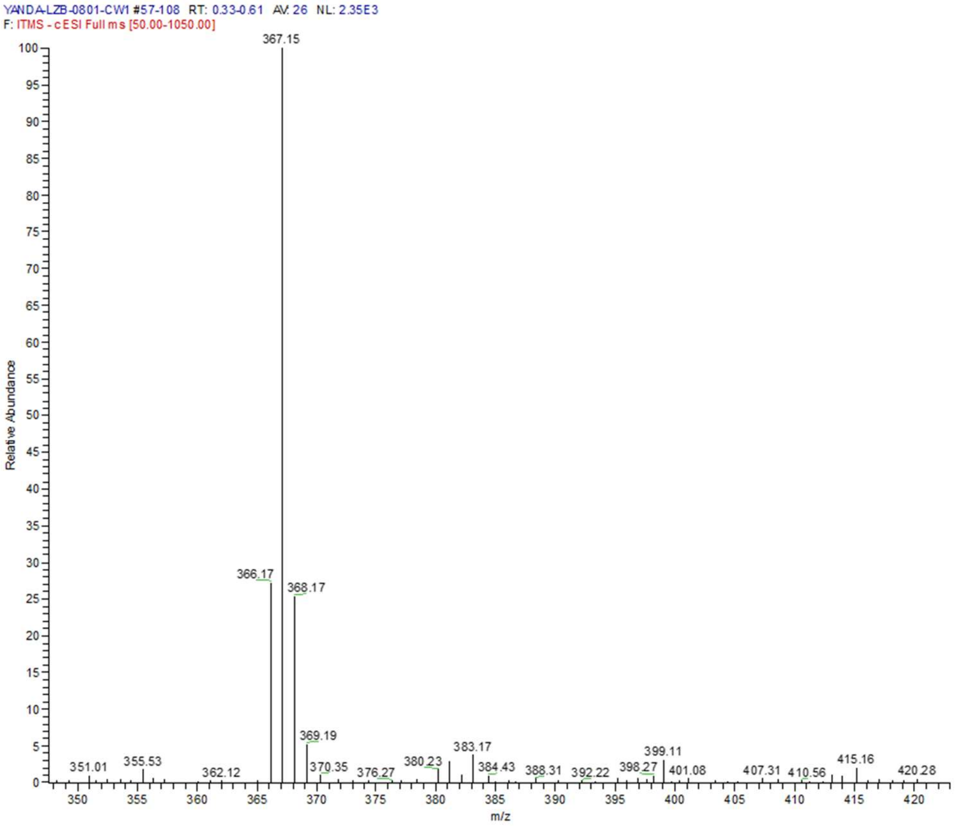

Figure S2 Mass spectrum of Compound 1

3. ¹H NMR of the Compound 2

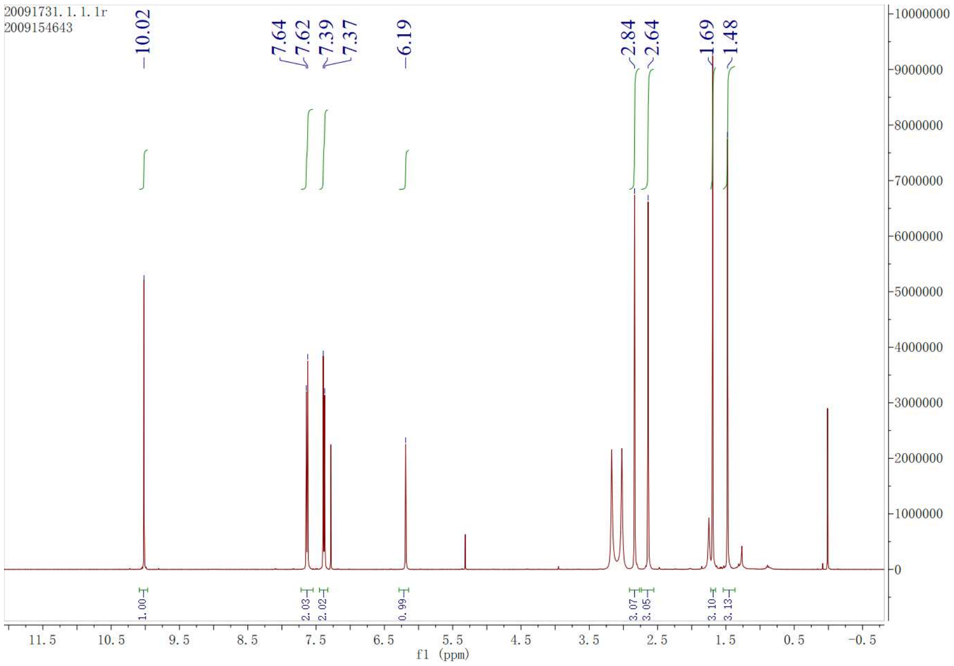

Figure S3 ¹H NMR of the Compound 2

4. Mass spectrum of Compound 2

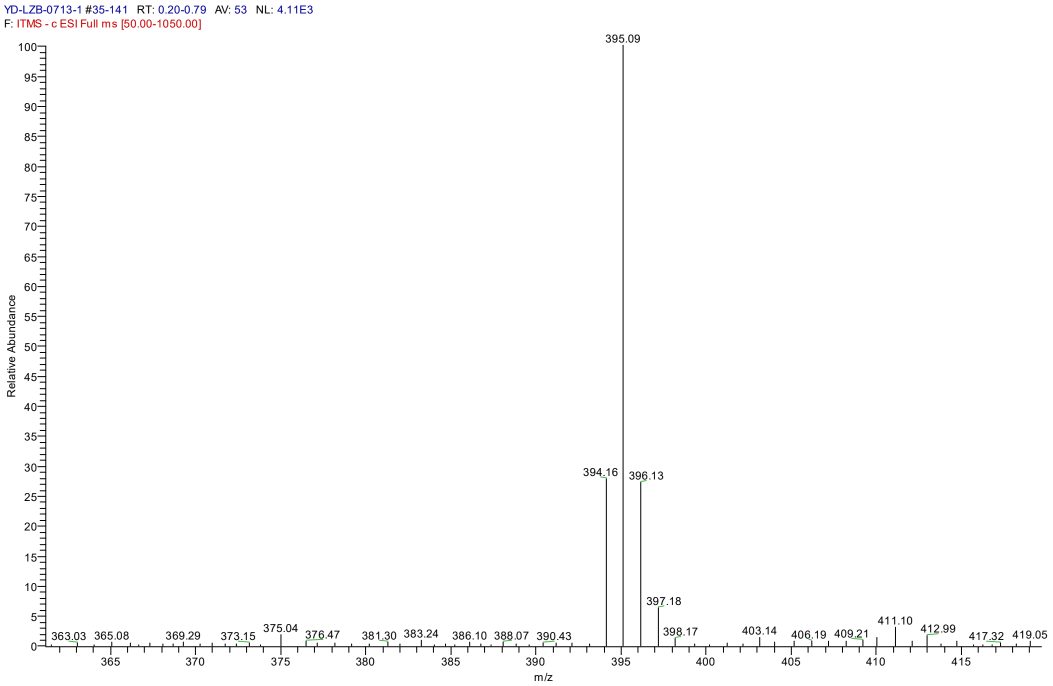

Figure S4 Mass spectrum of Compound 2

5. ^{1}H NMR of the compound fluorescent probe BODIPY-Y

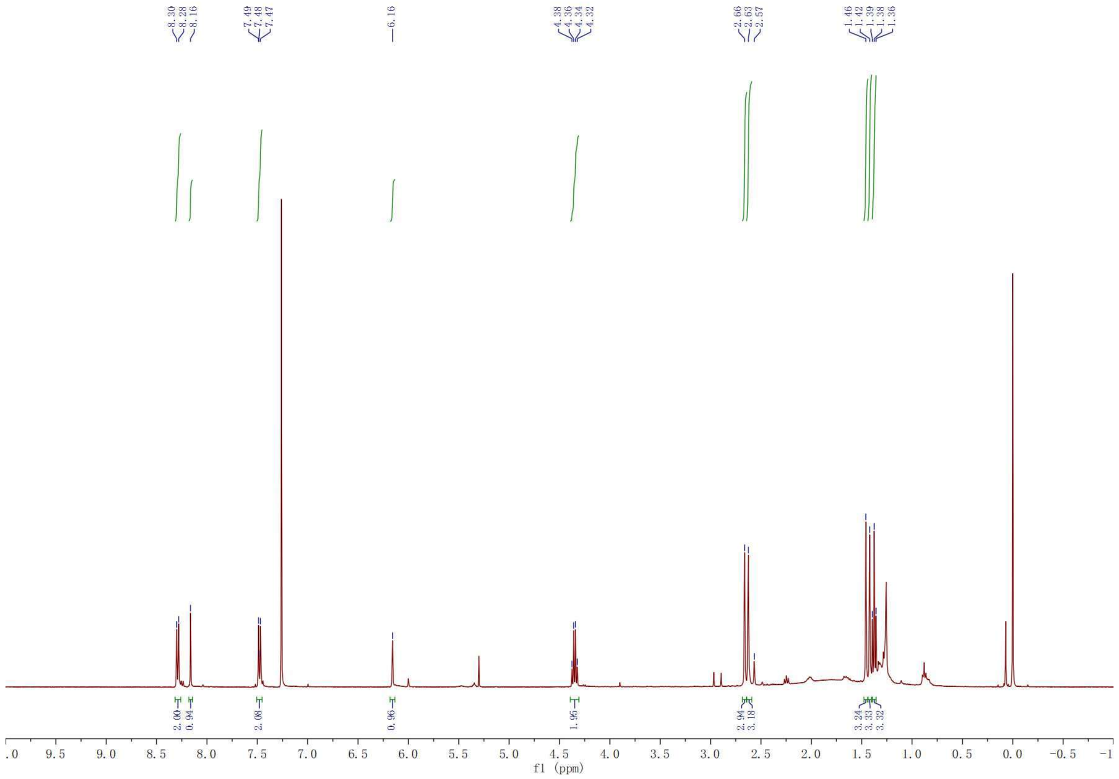

Figure S5 ^{1}H NMR of the target compound fluorescent probe BODIPY-Y

6. Mass spectrum of the compound fluorescent probe BODIPY-Y

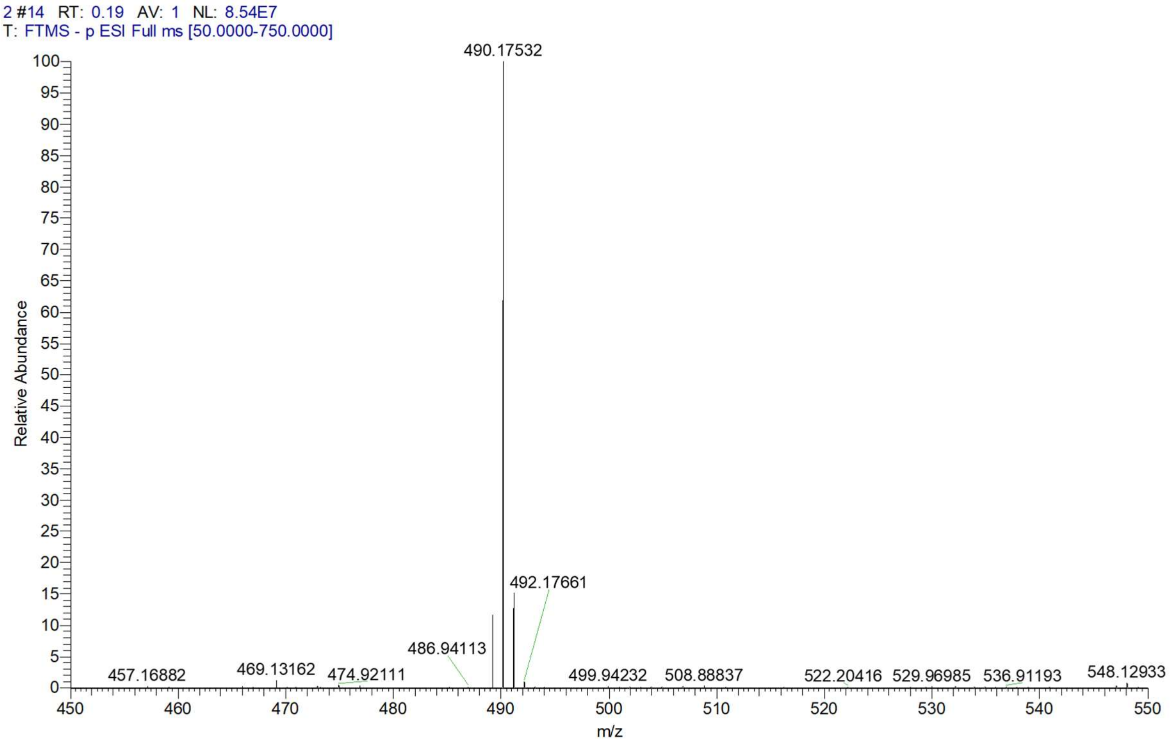

Figure S6 Mass spectrum of the target compound fluorescent probe BODIPY-Y

7. Mass spectrum of BODIPY-Y after reaction with SO_3^{2-}.

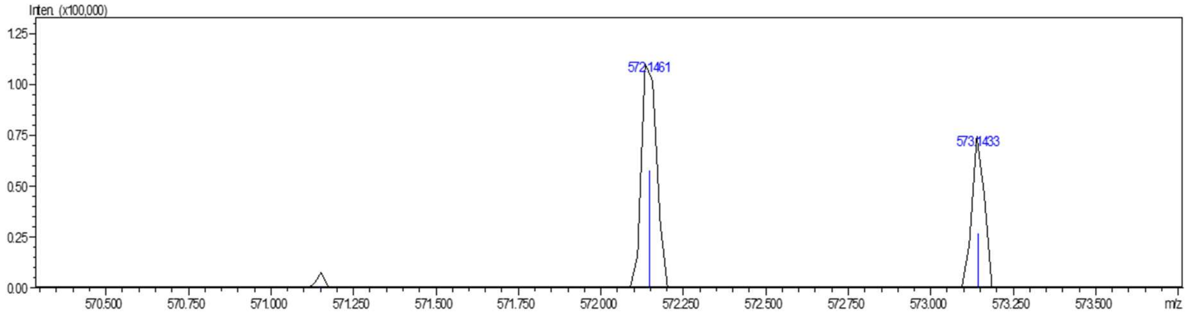

Figure S7 Mass spectrum of BODIPY-Y after reaction with SO_3^{2-}.

8. Comparison of fluorescent probes for SO_3^{2-}.

Table S1. Comparison of fluorescent probes for SO_3^{2-}.

Entry	Ref.	Fluorescent probes	LOD	Response time	Application
1	[43]	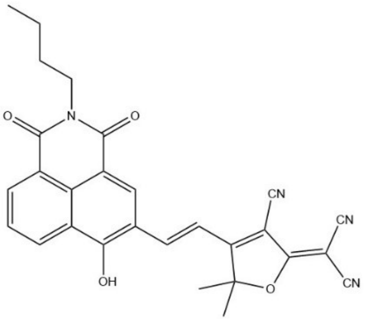	1.53 μmol/L	7 min	Living cells Imaging.
2	[44]	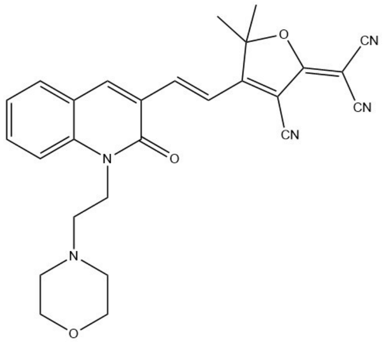	0.37 μmol/L	30 s	Living cells Imaging.
3	[45]	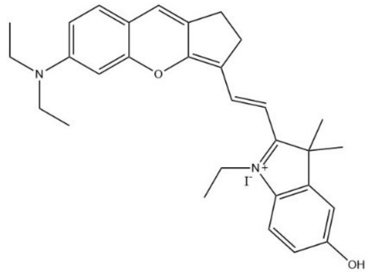	0.75 μmol/L	20 min	Living cells Imaging.
4	[46]	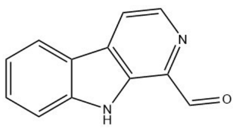	0.35 μmol/L	30 min	Living cells Imaging.
5	[47]	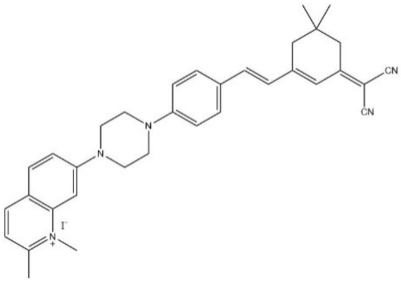	1.1 μmol/L	60 min	Living cells Imaging.

6	[48]	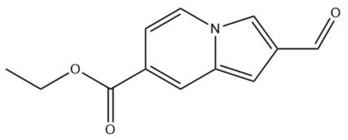	8.1 μmol/L	10 s	Living cells Imaging.
7	[49]	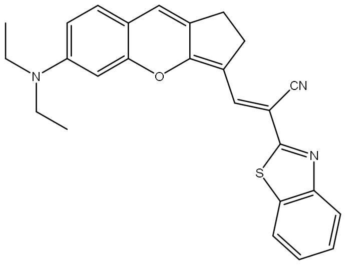	9.48 μmol/L	135 min	Food
8	[50]	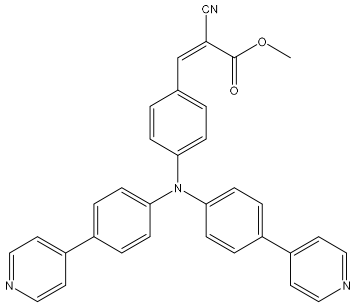	6.1 μmol/L	5 min	Living cells Imaging.
9	[51]	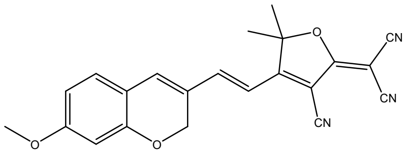	0.34 μmol/L	20 min	Living cells Imaging.
10	[52]	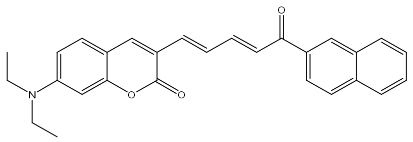	0.31 μmol/L	30 min	Living cells Imaging.
11	This work.	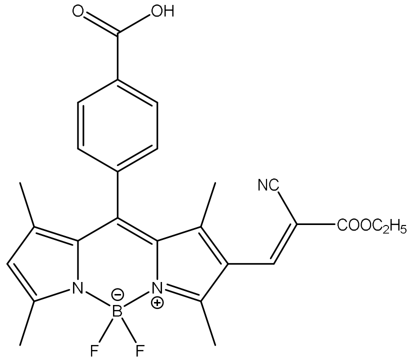	0.263 μmol/L	50 s	Food.
